# Neoliberal Ideology in France: A Qualitative Inquiry

**DOI:** 10.3389/fpsyg.2021.686391

**Published:** 2021-06-29

**Authors:** Lola Girerd, Catherine Verniers, Virginie Bonnot

**Affiliations:** Laboratoire de Psychologie Sociale, Université de Paris, Paris, France

**Keywords:** neoliberal ideology, governmentality, qualitative research, template analysis, France

## Abstract

This article adds to the existing literature on neoliberal ideology by investigating its content and contours in a context historically marked by statism. Very few studies in social psychology have looked at how neoliberal ideology transpires out of people's discourses and none have done so in such contexts. Yet, this appears necessary in order to better understand its actual influence and how it interacts with localized norms. Relying on a qualitative analysis of 32 semi-structured interviews and on the existing literature, we identified five central themes of neoliberal ideology in France: State prerogatives, competition, abstraction from institutional and social contexts, the entrepreneurial self and emotional management. Results suggest that the influence of neoliberal ideology transpires in the way people envision competition as something natural and motivating, in the way they distance themselves from their immediate and distant contexts, and in the way they value and engage in self-regulation while pursuing happiness and self-optimization. We also found that, in the French context, neoliberal ideology was not necessarily associated to the willingness to see the State step back and to the rationalization of all types of inequalities. Indeed, in the eyes of participants, the State largely remains the guarantor of public services and should ensure an equality of opportunity. This study highlights the value of relying on a qualitative approach to provide a rich and complex account of social realities such as ideologies.

## Introduction

For several decades, most countries of the world as well as international agencies such as the World Bank or the International Monetary Fund (i.e., the Washington consensus), have undergone a neoliberal turn (e.g., Navarro, 2007; Bettache and Chiu, 2019). Indeed, after the Chilean experimentation that started in 1973 (Araujo and Martuccelli, 2013; Barder, 2013), neoliberal policies have been increasingly adopted across all continents (Navarro, 2007), either encouraged by the Washington consensus (e.g., Morocco, Egypt; Hanieh, 2015; Argentina; Önis, 2006; Greece; Kretsos, 2012), by ideological endorsement (e.g., the two emblematic figures of Reagan in the United States and Thatcher in the United Kingdom; Schmidt, 2001; Prasad, 2006; Orléan, 2013), or by pragmatism (i.e., adapting to international economy and globalization; e.g., in France; Schmidt, 2001; Fourcade-Gourinchas and Babb, 2002).

Neoliberalism has been approached by several research fields and can be difficult to define (Birch, 2015; Venugopal, 2015). Its scientific and political use is even controversial [for critical discussions of the concept and its scientific soundness, see Dunn (2017) and Eriksen et al. (2015)]. However, and while keeping this complexity in mind, this paper will rely on a relatively consensual definition of its key features (Birch, 2015; McDonald et al., 2019). As an extension and intensification of capitalism (Springer et al., 2016; Ratner, 2019), it includes market-friendly policies such as the privatization of state-run agencies, the liberalization of capital investment and trade in goods, a focus on controlling monetary inflation, a deregulation of labor and markets, and the “marketization of society” through various forms of commodification (Harvey, 2007; Birch, 2015; Springer et al., 2016).

Such policies have concrete consequences, such as an increase in social inequalities and poverty (Harvey, 2007), and while our material environment does have psychological implications (Abrams and Vasiljevic, 2014; Ratner, 2019), the influence of neoliberalism exceeds that of its policies. Indeed, neoliberalism relies on and promotes an ideology that upholds such an economic and political vision and that shapes individuals to be well-functioning neoliberal subjects (Binkley, 2011a; Scharff, 2016; Ratner, 2019).

### Looking at Ourselves and Society Through a Neoliberal Prism

A central component of any ideology consists in describing a preferred way to distribute limited resources. Should they be distributed according to people's need or merit, in an egalitarian manner or some other criteria? Whom resources should be made available to? Are there groups that should be excluded from this operation? If so, for what reasons? As described in the Anglo-Saxon literature, a central tenet of neoliberal ideology is the perception that resources should be allocated based on individual merit (Zucker and Bay-Cheng, 2010; Bettache et al., 2020), which is closely related to the notion of individual responsibility (Baker, 2010; Pyysiäinen et al., 2017). Consistently, effort and competence should be the sole determinants of what one gets and only a competitive environment can allow that the most deserving wins. Likewise, competition appears as something natural (Gershon, 2011; Bay-Cheng et al., 2015). The State should ensure that competition be the golden rule in as many sectors as possible, between individuals and between firms and organizations (Foucault, 2004a; Pulfrey and Butera, 2013; Beattie et al., 2019). Moreover, because people should be held responsible for their lot, the State is excused for disengaging from certain roles, especially when it comes to inequality reduction, resources distribution or ensuring a safety net for the most disadvantaged (Harvey, 2007; Simon, 2016).

Neoliberalism is also considered as a form of governmentality, a mode of governance that goes beyond State control and that individuals exert on themselves (e.g., Foucault, 2004b). From this perspective, people are taught to govern and regulate themselves in a way that is coherent with neoliberalism (Brown, 2003; Mcdonald et al., 2007; Bettache and Chiu, 2019). This includes the shaping of individuals as entrepreneurial, calculating, competitive, responsible and happiness-driven subjects (e.g., Binkley, 2011b; Davies, 2016; Scharff, 2016). Accordingly, individuals are supposed to invest in their own human capital to maximize their chances of success on the competitive market (Becker, 1993). In fact, human capital represents the skills, knowledge and even health that individuals can foster for themselves, and mothers for their offspring (Salzinger, 2020), in order to enhance their market value (Burton-Jones and Spender, 2011; Arfken, 2018). The human capital approach thus offers an interesting account of people's increased reliance on self-regulation and self-transformation to adapt to the market in neoliberal times.

Neoliberalism fosters this mode of governance by promoting certain beliefs and values, by shaping people's aspirations, by psychologizing structural issues and by prescribing certain behavioral, psychological and emotional reactions (e.g., Larner, 2000; Beauvois, 2005; Pedwell, 2012), inasmuch as an ideology dictates how one should think, feel and react toward specific events (Ratner, 2019). For instance, research conducted in France has already brought some evidence that neoliberal ideology could hinder engagement in collective action aimed at addressing structural disadvantages (Girerd and Bonnot, 2020; Girerd et al., 2020). Yet, at this point more research is still needed to better grasp the content of this ideology, and thus its consequences. In fact, this research will focus on the ideological aspect of neoliberalism, as situated in a French context.

Indeed, neoliberal ideology can adapt itself to country-specific variations (Anderson, 2015; Arfken, 2018), which participates in its hegemonic power. Accordingly, the study of neoliberal ideology has to take those variations into account in order to better grasp the psychological and behavioral consequences associated with its deployment (e.g., Beattie et al., 2019), both at an individual and collective level. For instance, Harvey (2007) noted that the degree of penetration of neoliberalism into common sense within a particular context depended on the extent to which the notions of social protection, social solidarity, and collective responsibility were already embedded in that context. Now, in social psychology and to our knowledge, there is no account of the specific content of neoliberal ideology in a context of historical statism (i.e., in France). Therefore, the present study aims at filling this gap by investigating the content of neoliberal ideology in France.

### French Specificities

In France, neoliberal policies have emerged starting in 1972 under the (rightist) Minister of Finance Giscard d'Estaing (Foucault, 2004a)^1^. According to Fourcade-Gourinchas and Babb (2002) France begun to implement neoliberal reforms for pragmatic more so than ideological reasons. Indeed, it was believed that those reforms were necessary for France to adapt to the changing international economy in a context of European integration (Fourcade-Gourinchas and Babb, 2002). Importantly, this implementation was met with great resistance, as illustrated by the massive strikes of 1995 under the rightist Juppé government against public sector pension and social security reforms. For Schmidt (2001), this resistance can be easily explained by the lack of legitimizing discourses that would, at that time, convince the public of both the necessity and appropriateness (i.e., appealing to national values and identity) of neoliberal reforms. Therefore, the French citizens found little incentive to accept cuts in their social programs and public services and up until today, they mostly want to hold on to them (Le Figaro, 2018). Béland and Hansen (2000) also noted that “neoliberal ideology enjoy[ed] only a precarious legitimacy” in France at the time of their paper.

Moreover, France has been described as having a “large State” with tax revenues that are higher than those of most countries of the world, with a workforce working for the State as well as a welfare State that are larger than the ones of the United-Kingdom, the United States and Germany for example (Prasad, 2005). The French welfare state remained quite resilient even after the economic crisis of the 1970's because this crisis was not associated to the welfare state itself (Kus, 2006), which therefore remained relatively uncriticized at that time.

It is only under the Jospin (leftist prime minister) government of 1997 that a legitimizing discourse started being fruitful, in part by declaring that the promotion of social equity went hand in hand with economic efficiency. Interestingly, this discourse worked precisely because it managed to reconcile neoliberal reforms with the promotion of social equity and thus respected traditional social values of egalitarianism and willingness to care for the most vulnerable (e.g., Schmidt, 2001; Jetten et al., 2020). For instance, Jospin argued that while they would address the dysfunctions of the welfare state, they would not dismantle it (Schmidt, 2001). Indeed, even politicians who held economically liberal views remained very careful not to “question the principle of welfare” (Béland and Hansen, 2000). In a study conducted in France, participants -French university students- still rate the State as most responsible for reducing poverty (Krauth-Gruber and Bonnot, 2020), which suggests that the State is still credited with social prerogatives, at least among this sub-population.

While a form of resistance against neoliberal reforms still continues today, as illustrated for instance by the Yellow Vest movement that started in 2018 (Wilkin, 2020), or the strikes and protests of 2019 against the pension reform of Philippe's government (rightist prime minister under the Macron presidency), we do expect that years of legitimizing discourses concerning the necessity and benefits of neoliberal reforms would result in a shift in the degree of acceptance and endorsement of neoliberal beliefs and values. In addition, politicians are not the only ones resorting to the neoliberal rhetoric. It can also be found in the self-help literature for instance, and positive psychology in general (e.g., Rimke, 2000; De La Fabián and Stecher, 2017), as well as in the mass media (Nafstad et al., 2007; Guilbert, 2011). Also, neoliberal policies themselves affect people's mentality (Ratner, 2019). The size of the French State has indeed been reduced throughout the past decades while several sectors, including banking, insurance, energy and oil, have been privatized and now answer to the market logic (Prasad, 2006). This may change the way people relate to the State and how they think about its prerogatives. Moreover and to give just one other example, permanent working contracts have become rarer whereas temporary contracts have flourished (Papinot, 2009). This helped construct flexible, solitary, interchangeable, hyper-productive and non-unionized workers (Papinot, 2009). These are all ways to distill neoliberal governmentality.

In sum, the French context is marked by two opposite forces: on the one hand a historical and normative attachment to social values of egalitarianism and support for the less well-off (Jetten et al., 2020) that is related to an attachment to the welfare state, and on the other hand a shift toward neoliberal practices, beliefs and values. This research aims to better grasp the frontiers of neoliberal ideology in this unique context.

The goal of this paper is indeed to offer an empirical account of neoliberal ideology in a country historically marked by statism. Accordingly, we believe that a qualitative approach is best equipped to tackle this question as it reduces the risk of forcing a neoliberal prototype onto participants, instead leaving more room to a nuanced, complex and even contradictory version of neoliberal ideology. That is why we relied on data obtained through 32 interviews and that helped us identify the themes that best characterize neoliberal ideology in French people's discourses. Similar endeavors help document elements of a neoliberal discourse in countries such as Australia (Baker, 2008, 2010), the United Kingdom and Germany (Scharff, 2016), Chile (Araujo and Martuccelli, 2013), and the United States (Morris and Korobov, 2020). Extending this literature, this research aims at identifying the dimensions of neoliberal ideology in France, and how its shapes people's discourses.

## Methods

### Participants

Thirty-two participants (17 women) were individually interviewed for the study (Table 1). Sample size was based on the saturation criterion while we aimed to gather a heterogenous sample. Those elements were also balanced against the time and resources that were available. The mean age of participants was 29.42 years old (*SD* = 8.08; range = 20–50). Recruitment was limited to people under 50 because we wanted participants who had lived under neoliberalism for most of their life. All participants were of French nationality. Note that the participants in our study are more educated overall than the general population (in 2019 only 23.2% of the general population had higher education equivalent to 2 years after high school or more; Institut National de la Statistique et des Etudes Economiques, 2020; see Table 2). Of the 29 participants who positioned themselves on the (1) extreme left to (9) extreme right continuum (data is missing for one participant and two participants refused to position themselves on this continuum), the mean score was 4.03 (*SD* = 1.59), thus bending slightly to the left side of the spectrum.

**Table 1:** Socio-demographic information of participants.

**Participant**	**Gender**	**Age**	**Occupation**
1	Male	42	Regional referent for energy transition and wood broker
2	Male	50	Manager
3	Female	29	NA
4	Female	22	Beautician
5	Female	20	Psychology student
6	Female	22	Fashion student
7	Male	21	Intern-Business development and operations
8	Female	25	Laboratory technician
9	Male	23	Consultant in organization management
10	Female	24	High school French teacher
11	Female	30	Accountant assistant
12	Male	38	MLM entrepreneur
13	Male	34	Freelance and Mediapost distributor
14	Male	40	Temporary research and lecturer assistant
15	Male	23	Political counselor
16	Male	29	Administrator in a portfolio management company
17	Female	25	Student in political science
18	Female	23	Student in industrial engineering and project management
19	Male	24	Student in political science
20	Female	20	Psychology student
21	Female	24	Law student
22	Female	28	(Unemployed) creating a business
23	Male	NA	Military
24	Female	40	Engineer
25	Male	40	Barrista
26	Female	24	Computer engineer
27	Male	45	Business owner
28	Female	30	Unemployed
29	Female	32	Social worker
30	Female	34	(Parental leave) school teacher
31	Male	27	Sound technician/engineer
32	Male	24	Computer science student

**Table 2:** Description of the educational attainment of participants.

**Educational attainment**		
2 years after high school or less	3 or 4 years after high school	4 years or more
12 (38.71%)	8 (32.26%)	12 (38.71%)

Participants' recruitment followed the snowball procedure, starting from a convenience sample recruited by email or telephone directly or through intermediaries, while targeting under-represented segments of the population as we went along with the interviews. The interviewer did not share a proximate relationship with any of the interviewees but knew some of them prior to the interviews. All interviews took place between December 20th of 2018 and February 26th of 2019. Note that this was during the Yellow Vests protests, which may have influenced participants' responses. In order to minimize social desirability, we presented the study as an inquiry on people's perception and feelings about societal issues. We carefully avoided mentioning the terms “neoliberalism” and “ideology” or any statement revealing the political views of the researchers. An overview of the results was later addressed to all participants.

### Procedure

The first author (i.e., a Caucasian female PhD student in her mid-20's) conducted all interviews. Twenty-one were face to face, the remaining were online Skype interviews. At the beginning of each session, participants were introduced to the context of the study and were asked to give their consent regarding their participation to the study and the audio-recording of the interview. During transcription, references to people's names as well as specific locations were anonymized. Participants were told that the interviews would be made anonymous and that there were no right or wrong answers to incite them to speak freely. The research was conducted in accordance with the World Medical Association's Declaration of Helsinki, but we did not seek the explicit ethics approval as it was not required as per our institution's guidelines and applicable national regulations.

The interviewer relied on a semi-structured interview guide with open-ended questions that was adjusted after a pilot study conducted with 11 participants. The purposes of the pilot study were to improve the original interview guide and to train the interviewer. The interviewer prompted participants to elaborate on their answers or to define specific concepts relevant to the research question. Participants were asked about their objectives in life, the obstacles that they may encounter (inspired from Baker, 2008), the importance they gave to their relationships with other people, about notion such as independence, freedom, their perception of inequalities within the French society and of potential solutions to remedy them, about government intervention and competition.

The interviews lasted between 23 and 175 min, with an average of 51 min (*SD* = 28.59). Upon completion of the interviews, participants were debriefed and were given the opportunity to express their thoughts and feelings about the questions they had been asked and to add anything if they wanted to. Finally, they completed socio-demographic information including sex, age, and political orientation and were thanked for their participation.

### Data Coding and Analysis

Atlas-ti 8 software was used to analyze the interview transcripts. We relied on a template analysis, a type of thematic analysis, to analyze the data (Brooks et al., 2015). Indeed, it allows for a flexible but structured analysis combining both bottom-up and top-down approaches. It also allows the researcher to start the coding process with a priori themes derived from the literature and that help drive part of the analysis, while leaving the possibility for the themes to be dropped or refined during the coding process. Moreover, this method allows to find patterns in all the dataset, which matched our goal not to look at the data of each individual separately, but at the aggregated data of all participants.

In the first step of the analysis, we created themes based on the literature (Baker, 2008, 2010; Bay-Cheng et al., 2015; Arfken, 2018; Beaumont and Kelly, 2018; Rottenberg, 2018; Teo, 2018; Adams et al., 2019). They helped us guide the coding of a subset of the data comprising of eight interview transcripts. Based on this first analysis, a first version of the coding template was elaborated. In the second step and applying this template, the first author refined the coding of the eight transcripts. To assess inter-coder agreement, we applied the method and steps given in Campbell et al. (2013) and the calculating method provided by Miles and Huberman (1984). Accordingly, a second researcher (i.e., the third author) double coded three of the transcripts and the two coders discussed their possible disagreements with the coding. Following the discussions, inter-coder agreement reached 99.74%. Discussion between the two researchers also led to a refinement of the first coding template. In the third step, the first researcher applied the template to the rest of the dataset and revised it when necessary in an iterative process.

We agree with Brooks et al. (2015) that the epistemological position of our use of the template analysis should be made explicit. In this study, we endorsed a “subtle realist” standpoint (Hammersley, 1992), which assumes that researchers are necessarily influenced by their own position in the social world, while maintaining the belief that the research process can still give access to phenomena that exist independently of the researchers (Brooks et al., 2015).

## Results

**Figure 1:**
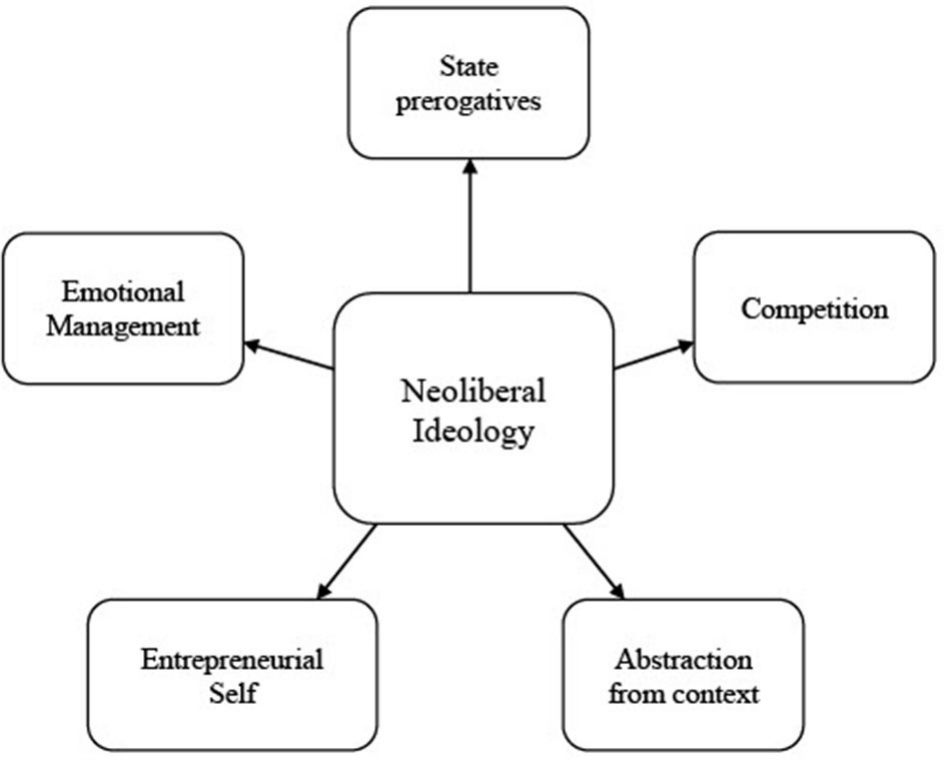
Dimensions of neoliberal ideology in France.

The analysis led to keep the following themes: (1) State prerogatives, (2) Competition, (3) Abstraction from context, (4) the Entrepreneurial Self, and (5) Emotional management (Figure 1). More information about the subthemes, their definitions and prevalence is available in Supplementary Material 1.

Because we did not expect, and did not find participants to match the archetype of a “neoliberal subject,” we remained open to incongruences and even contradictory responses. However, because of limited space, we will focus on what is coherent with a neoliberal orientation and what can reasonably be considered a French specificity. Complementary analyses can be found in Supplementary Material 2.

### State Prerogatives

Endorsement of neoliberal ideology in the United States has been associated to the belief that the government was too invasive and should not interfere in people's lives, including to address social inequalities (Bay-Cheng et al., 2015). Considering the French historical and cultural attachment to the welfare state, we expected this dimension to differ. Moreover, the French historical attachment to the equality ideal (as in its motto: “Freedom, Equality, Fraternity”) may also influence the way participants conceptualize inequalities, the extent to which they rationalize them and thus how they think of the State's obligations, or lack thereof, in relation to them (Jetten et al., 2020).

Indeed, social inequalities do exist in France and have actually increased in the last decades (Piketty, 2013). Importantly, merit related discourses are prevalent in France (Tenret, 2008) and they hold the intrinsic power to diminish the expectation that the State should fight income disparities. As such, those discourses are at odds with egalitarian measures that inherently question the merit principle (Darnon et al., 2017).

Consistently, several participants expressed that resources should be allocated based on individual merit and thus that inequalities that resulted from this mode of distribution were unproblematic (Duru-Bellat, 2019):

It's normal that in a country people don't all have the same salary or earn exactly the same amount, people haven't put in the same effort, or completed the same studies and not everyone has climbed the ladder in the same way. (#18)

At the same time, this endorsement of meritocracy seems contradictory with the egalitarian norm that has historically coexisted in France. Interestingly and as illustrated above, we almost never found this egalitarian expectation, suggesting that this norm does not, or no longer, extend to economic resources distribution. This is consistent with Schmidt's (2001) observation of a shift in the French Socialists' discourses in the 80's from one on efforts to equalize income disparities to one that focused on solidarity and which was thus more accepting of such inequalities. Consistently, we came upon an unthreatening conciliation between meritocratic demands and the norm of equality: the focus on equality of opportunity. Indeed, the vast majority of participants was particularly critical of the existence of inequalities of opportunity, more so than they were of inequality in resources. In this regard, they agreed that the State should guarantee, through public education for instance, equal opportunity:

Definitely school. Completely reform schools, you have to put money into schools umm and upgrade the system, well the importance of, of… knowledge. Personally, well I think there really are things which can remove umm alleviate inequalities of birth and well you see umm that's it, knowledge, culture, things which can umm. (#10)

However, even critiques of inequalities were sometimes framed in terms that were consistent with neoliberalism. In the following quote for instance, a better access to professional and educational opportunities was not described in terms of social justice but as a way not to waste promising human capital (Becker, 1993), in order for our society to be more efficient:

I'm not saying that BTSs [Brevet de technicien supérieur: Advanced Vocational Diploma] aren't good, but 2 years afterwards he's going to be on the job market, whereas he has maybe the potential to do amazing things, get into fantastic prestigious schools and thanks to these schools and the networks they offer, be able afterwards to have ideas which will help you progress and have… I think that there's a potential which, there's a lot of people who have incredible potential which isn't exploited, because of that, because of the fact that they haven't been given the opportunity to access education that allows them to fully express themselves. (#7)

Overall, beyond participants' willingness to see the State ensure an equality of opportunity, and contrary to the United States (Bay-Cheng et al., 2015), they rarely agreed that the State should not intervene in people's lives. Consistent with traditional equality-based values, in general we did find an expectation that, at a minimum, society shouldn't be too unequal and that the State had a role in the matter. The vast majority of participants expected the State to provide, at least minimally, for its citizens. This largely transpired in the way they talked about public services:

As an economist umm (laughter) we need public services umm public, not privatized… And in fact that depends in which fields, but for example public utility fields such as transport, you'd say water umm, well the management of water, electricity, you know it's more efficient when it's managed by the state. Because it's something that's communal, so already when it's privatized… umm you're sure (emphasizes) that you have inequality in terms of access. And when it's the State, no, well less, less so (emphasizes). (#15)

Following the idea that the State should intervene to reduce inequalities, public services were often preferred by participants over privatized companies notably because the latter were perceived as increasing inequalities:

But on the other hand, I think for example about SNCF [Société Nationale des Chemins de Fer Français/France's national rail service] and all that, it's to do with public service in a way as well because, it means that there's certain areas which will have fewer services or lines which will be cut, which is already the case and everything. That can be useful for the community, so I think that those which are priv, umm which are not yet privatized, that's in fact why, there's a sort of idea of common interest, it'd be better to keep it in fact, that it be nationalized so that the common interest is put before financial interest. (#30)

Those quotes illustrate the expectation that some services should be available to all, independently of one's resources. We see here that the endorsement of meritocratic principles is not limitless and does not include access to services such as energy, transportation, education or healthcare. Many participants did not apply a market rationale to all areas of society and especially not to those that are still managed by the State today:

Umm we're quite lucky in the sense that umm, umm that efforts are made to try and help families who are less privileged than others, there's social assistance which provides the minimum assistance, there's grants to umm allow some children but who have anyway had access to certain opportunities to access higher education [hmm]. (#18)

As mentioned in the introduction, supporting at least some level of equality is considered normative in France and is part of the French “myth of equality” (Sénac, 2017). Several participants did perceive this norm and a societal wish to address social inequality:

No, no it [France]'s not egalitarian […] Umm… but it wants to appear egalitarian, anyway we have a society which sends out messages about it, well of course it's umm the basis of our society, we want society to be as egalitarian as possible but no, it isn't. (#1)

However, some participants did describe positive aspects of privatization. For instance, that it would reduce the deficit of the State and that it would lead to more efficiency, for instance “the SNCF, personally I thought it was good that the, the rail network was finally open to competition, that's what helps to improve the service, in my opinion” (#18). This rhetoric has indeed been relied on by politicians when justifying austerity measures and cuts in public services (e.g., Louis, 2019).

In sum, overall neoliberal ideology does not (yet) translate into an expectation to see the State completely disengage from its social duties. However, the endorsement of meritocracy certainly reorients people's attention toward issues that do not threaten the heart of neoliberalism. Moreover, even in maintaining that the State should guarantee equal opportunity, participants implied that it should in fact guarantee a fair competition, which is another central component of a neoliberal worldview.

### Competition

Since the competition game is not perfectly fair, because starting with more economic, social, symbolic, cultural resources insures greater chances to gain even more resources, competition eventually helps the dominant class to remain in power and to keep absorbing more resources. However, because neoliberal ideology partly serves the purpose of obstructing the perception of injustice and oppression (Ratner, 2019), people are led to believe that competition allows for individual mobility given that one puts in sufficient effort, that it is part of human nature to be competitive, and that it is inevitable and beneficial at the societal level. Such beliefs as well as a perception of the competitive aspects of neoliberalism can be found in our interviews, for instance “in the context of competition in, in business, of course you have competitors. You always want to be a business which prospers, which produces more, which becomes even better than its competitors. Of course, that's obvious.” (#27)

Competition also appears as self-evident and unavoidable at an inter-individual level, “I don't necessarily try to compete, well yes of course we're in competition with everyone but, well, that's how I see it.” (#32)

Moreover and because neoliberalism colonizes every aspect of one's life (Teo, 2018), it is not surprising that competition too, would affect more proximate social relationships:

Depending on who you're friends with, there can be competition within your circle, because automatically, in your social circle, or even in the family as well there can be competition. Well, seeing that it's a question of perception, you can have competition everywhere in fact. Seeing that competition is also a bit like, it can be: “am I better than you or not, have I had more success.” (#30)

Interestingly, despite the fact that participants did perceive some environments to be competitive, they also surprisingly often described competition as a preference or as depending on a person's perception. Above, the participant explains the pervasiveness of competition by the fact that it comes with one's perspective “seeing that it's a question of perception, you can have competition everywhere.” Similarly, competition was sometimes described as a personality trait, “I'm fairly umm, competitive by nature, it's in my personality.” (#31)

However, despite this expansion of competition, it does not appear necessarily normative to discuss it. This may be related to the normative negative connotation of individualism (i.e., in the sense of prioritizing one's own goals over collective goals) and selfishness in the French context (Dubois and Beauvois, 2005; Springer et al., 2016). This may illustrate a tension between neoliberal injunctions and localized norms:

Last year I was taking a competitive exam, but you see, me, I always have to, in fact especially with my friends, I'd never tell them, I sound awful (laughter). In fact umm I don't say it umm but yes, I have to, me, I like, you see I really like being better than my friends y'know. (#10)

By saying “I sound awful” this participant acknowledged that she knew she was stepping out of what is normatively acceptable: proximate relationships should not fall under the prism of competitive practices.

Relatedly, competition was not always perceived as something positive, “me, frankly I went to an elite middle and high school where there was competition umm it was scary though it shouldn't have been, and I think it did me a lot of harm” (#29), or again:

The disadvantages, the problem is always wanting to do more, and well sometimes you put your own health at risk y'know, either physical or mental. [Do you mean constantly wanting to do better?] Yes, yes, always striving to go further. Yes, in competition. (#26)

Therefore, we see here that competition can be perceived as a threat to one's self-esteem and well-being. Participants sometimes also described it as being antagonistic with another injunction of neoliberal ideology, namely happiness. This may stand as another point of tension in neoliberal ideology:

It can also hold you back because you, you focus on how others are doing and sometimes you can tell yourself that you're way off that, and you forget that what counts as well is your own personal development. (…) If you spend your time like that in fact you actually forget to be happy, in fact. (#30)

But despite those negative aspects, many participants did agree with the neoliberal rhetoric of competition as a crucial source of motivation, “but all the same, competition is always motivating I find. Without competition umm, I don't know whether I would want to strive for anything” (#6), and as a source of personal growth and progress:

If it's for looking for a work placement that will umm highlight more the skills that I haven't focused on up till now [hmmm]. It will also perhaps develop the skills I need, so doing extra courses umm learning a language umm traveling to gain experience. (#18)

Participants also consider competition as a source of innovation “competition as long as it's used to stimulate people among themselves to find new ideas and move things forward in the right direction, it's great.” (#31). A discourse where the self becomes a product to sell in the competitive market, “the advantage is that it forces us to be the best we can, to market ourselves the best we can” (#11).

As we have seen through those first two dimensions of neoliberal ideology, the wish, in principle, to see the State ensure a fair competition and stay in charge of public services, was not incompatible with the perception that individuals could remain largely unaffected by their context. This was mainly illustrated by the belief in meritocracy and the belief that competition also depended on one's perception or personality. The next section will focus on this perceived abstraction from context.

### Abstraction From Context

According to Adams et al. (2019), this “radical abstraction” from social and material contexts is partly the result of globalization and the increasing spatial mobility. While this permits a greater access to non-local opportunities, it also detaches people from their local communities, especially when local solidarity is effortful (i.e., when it demands time and personal investment). This theme thus describes the observation that people sometimes see themselves as being detached from (i.e., uninfluenced by, or unaware of) their material, social, ideological and normative environments (e.g., Baker, 2008; Brown, 2016).

#### Abstraction From Structural/Institutional Contexts

This sub-theme reflects the relative detachment that some participants express vis-à-vis institutional and non-institutional structures. For instance, some participants believed that they were independent of the State because they did not rely on public aids:

Well personally I've never needed to have too much umm… to do with the State. umm I'm, I'm a particular umm example, I've always got by, I've never asked for social support. (#12)

This underestimates the influence that the institutions have on our lives. Indeed, we interact with the institutions in various and numerous ways throughout our lives.

Additionally, we found a relative abstraction from large social structures. For instance, and echoing findings related to the theme of State prerogatives, we sometimes noticed an emphasized attribution of inequalities to individual merit, rather than being structurally determined inter-group inequalities. In the following quote, what economically differentiates between women is their ability to work hard and thus have access to well-paid jobs through their individual efforts:

Well it's a bit about merit in my opinion. There'll be inequalities but it's the same, it'll be based on merit and there'll be, there'll be women who have worked harder to get a better paid job than another woman who has worked less. (#21)

Indeed, several participants placed the emphasis on the individual rather than on groups' memberships to explain one's access to specific resources. When asked about obstacles that could hinder people's success, many participants answered with individual-level factors, “because they aren't cut out for it. Just like there are sporty people, there are people who are more intellectual” (#32), with no mention of the influence of one's gender, social class, race or sexual orientation for example.

Similarly, the term “inequality” was often framed as an a-political, inter-individual issue, which renders the term “equality” meaningless, if not unwarranted and undesirable:

Well, in reply, personally I don't like that word egalitarian too much, because, because, we can't be equal, we're all dealt a different hand right at the start, we all start from different places and umm….and so saying that society is egalitarian with people who are different, you can't be egalitarian. (#30)

In the quote above, “equality” is described as erroneous and impossible to reach because people are different. This implies that being equal means *being* the same and not *having* the same. And while disparities in resources can be debated politically, inter-individual psychological, biological or physical differences are a lot less politically debatable.

Personally I think that we shouldn't all try to be equal. umm…If we were all equal, all the same umm, if you think like me, if you react like me, what's the point, what's the point in discussing it. I don't see the point in everyone being equal. (#2)

In this quote, seeking equality would render inter-personal interactions uneventful and dull. Here too, the political force of the term “equality” vanishes as it ceases to embody a political vision referring to the distribution of resources, to equal rights or equal moral worth and becomes an imaginary project of uniformization. Consequently, it may leave a conceptual void to think of what equality entails in a political sense. Several participants preferred the term “equity” partly because it can be reconciled with the notion of merit:

I'm not really in favor of the term equality, I think what's important is to have equity rather than equality so umm, […]. (#3)

When participants did mention group-based inequalities such as those based on social class, gender or race, the source of inequality was still sometimes situated at an individual level. It emphasizes the difficulty or counter-normativity of relying on structural explanations for the existence of inequalities (Beauvois, 2005):

It's like there's racism in France but I think that, I dare to hope that the majority of French people aren't racist. In the end it's got a lot to do with education, people are racist because they haven't… through ignorance mainly. It's like hate, it's like anger, it's like violence, it's due to the ignorance of the world around us. Same for equality between men and women. (#9)

Here, racism and sexism are the business of some racist and sexist individuals who are uneducated or ill-educated. In the same vein, the following participant described social class inequalities as being the result of some people' selfishness, “umm that depends on attitudes as well, you know there are people who want to monopolize everything and don't share for example” (#13).

When participants blame ignorance or selfishness for inter-group inequalities, they partially leave the institutional structures off the hook. Moreover, when participants mentioned solutions to reduce inequalities, they also sometimes described individual-level solutions, like “training courses, teaching women how to be business leaders for example” (#26).

Thus, people should act upon themselves, acquire new skills, more so than try to change the structures allowing for such inequalities to exist and persist. This once again abstracts individuals from the structures in which they evolve. It locates the responsibility to solve disadvantages on people themselves, and more often on disadvantaged groups' members (Kim et al., 2018), as in the example above (#26).

There's two things [to reduce inequality]: there's actually a material side so a better distribution of wealth but it's dangerous to do that (…) for example there was a baker, then there was a guy who worked in a factory. And then, they won 12 million, two million [on the lottery], it had all gone in 2 years because they actually said to themselves well, I'm going to enjoy myself, I'm going to, and in the end, they had nothing, at the end of 2 years they had nothing left. Why? Because they didn't have any mental process telling them; err, I am going to create something with this. They have a way of thinking based on direct consumption, umm direct pleasure without actually thinking of the long term and making things grow, or asking themselves the question; how can I keep it, how can I actually make it grow and perhaps help others, what can I do constructively with that so that it doesn't all disappear? And so it's just not enough to give resources and means to people, you also have to teach them to, with that, how they can make it profitable, what can they do to get to grips with it, what is the process to actually make it grow in fact. (#22)

For this participant, members of lower social classes mismanage their finances and are described as unable to be future-oriented and be productive neoliberal agents. They should therefore learn how to think and act as such, otherwise, resources are simply wasted on them. Despite her admission that society should better allocate resources, she still establishes the neoliberal way of managing one's life as the ideal horizon, prioritizing changes in people over changes in the structures (Kim et al., 2018).

#### Abstraction From Social/Normative Contexts

Besides the abstraction from the institutional and structural contexts, we noted a willingness to consider oneself as being detached from social norms. The promotion of individuality, of the belief in free will and choice prompt people to perceive the choices they make as personal, self-determined and unique and thus to refuse the idea that they could fall prey to conformism (Rimke, 2000; Teo, 2018). This manifested itself when participants expressed their detachment from or even opposition to specific social norms, “I think it's very different from my view. My personal view. So I think that succeeding in life, for French society…? [hmm, hmm] Succeeding in life for French society means, completing your studies, studying at a prestigious university” (#9)

By saying “it's very different from my view,” this participant explicitly positions himself in opposition to the subjective norm he is about to describe. In another example, a participant describes two antagonistic normative injunctions concerning the role of mothers. One where mothers are supposed to stay at home, care for their children and take care of the house and a more recent norm where mothers should keep their job but end up missing out on their children's education^2^. In the end, she concluded that she'll “do which suits [her]” (#20), therefore affirming her own individuality and choice beyond those norms.

Besides distancing themselves from social norms, participants also expressed forms of detachment from proximate social relationships. This transpired in the way some participants defined the notion of independence: “emotional independence means not being attached to anything (corrects herself) to anyone, or you mustn't be in love, mustn't long to see someone, mustn't need or miss anyone, that, that's emotional independence” (#5), or:

I'd say that for me independence is also synonymous with autonomy. And who will be able umm…to find the resources to, to get by all on their own, say…[…] Being dependent […] that's someone who will always ask other people for things, they won't be able to manage on their own. (#30)

This is contrary to research stating that we are social creatures with social bonding needs (e.g., Baumeister and Leary, 1995). Neoliberal discourses indeed uphold the image of a self-defining, self-sufficient individual who cannot let relationships or social solidarities get in the way of their personal accomplishments or wishes (Teo, 2018; Ratner, 2019). Thus, it is not surprising that social (inter)dependence, emotional attachments and the mere fact of relying on others for help, advice or support were often described as something negative, undesirable and as a source of vulnerability. Another participant described how “the fact that they are very close, they support you but that influences you as well. And so you sometimes have difficulty listening to your inner voice y'know. And that that's an obstacle.” (#9). Again, the following participant, by saying “it's something I'm working on at the moment,” expressed her desire and effort not to rely as much on her partner for help or support and be less “dependent” on him:

When I'm part of a couple I tend to rely a lot on my partner, and sometimes umm… ask him to do things which I could do myself and be a bit dependent, I get the impression. It's something I'm working on at the moment (laughter). (#30)

Interestingly, one way through which participants reframed the -almost- universal need for satisfying social relationships that they might actually experience (Baumeister and Leary, 1995), is to consider it as a personality trait or personal preference. Here too, neoliberal ideology feeds the image of a unique self that does not share basic human psychological needs. The participant below described this need as reflecting her personality as a sociable person. While she hinted at a shared experience “your personality is shaped by being with other people,” she ended up going back to a personal desire for authentic and healthy relationships. Therefore, participants tend to reduce a “universal” experience to an individual one:

I'm quite umm, quite sociable, I… I need to have people (laughter) around me, well not, not always being with people but umm…. but it's important for me to have the ability also to rely on, [huh] on other people and then I find that rewarding, anyway, your personality is shaped by being with other people and umm and that's it so umm yes, it's important for me to have, relationships which are umm umm healthy, genuine. (#29)

We have seen that social dependences were sometimes seen as sources of vulnerability. Going further, the following participant described how being uninfluenced by the environment is the key to success because it allows one to be unaffected by external constraints and negative events:

[those who don't succeed] are fragile in the sense that umm they depend on an external environment […] For me people who don't succeed at school are people who are dependent. On something. For example on their parents, or…their loved ones… and what they tell them to do or not to do etc. (#15)

By describing dependence to one's context as a failure or a weakness, this participant not only denigrated people' sensitivity to their material and social environments but also implied that abstraction from them is possible and desirable. The following participant held similar beliefs, implying that remaining unaffected by “external conditions” was feasible, in saying that “independence is when you don't depend on external conditions, well when external conditions don't influence umm our state of mind in fact, our state of being.” (#22)

Participants often seemed conflicted when they tried to assess their own level of independence and many still tried to maintain an image of themselves as independent individuals. They often do so through downward social comparisons or by mentioning the effort they deploy to be -more- independent. This highlights the normativity of such an injunction but also points to another zone of tension where the neoliberal discourse does not reflect what people actually experience in their daily lives.

So far and consistent with the literature on neoliberal ideology, we have seen several ways through which participants tended to detach themselves from their structural, social and normative contexts. We will see now that this perceived detachment is rendered possible by the construction and promotion of an entrepreneurial self notably through the reinforcement of the norm of internality (Foucault, 2004a; Beauvois, 2005; Dubois and Beauvois, 2005).

### Entrepreneurial Self

Indeed, the entrepreneurial subject is one that is largely abstracted from their context and is autonomous, responsible and self-determined. One that owns up to their failures, successes, assumes responsibility for their choices and expect others to do same:

Strangely I think that the obstacles, we put them there ourselves a bit, […] personally I perhaps underestimate sometimes what I can do, or perhaps I'm sometimes frightened of saying, when you have a slightly comfortable situation because that's what you know and finally well when you don't know something it's more complicated to launch into it. So it's perhaps more me doing it. (#3)

Umm err in the context of school and work err what can stop us doing well err one thing I think already err motivation, that can do it eh: someone who is more motivated than someone else, err the one who is more motivated is bound to succeed more. (#4)

In both cases, participants attribute difficulties to internal causes, lack of self-confidence in one case and lack of motivation in the second. Understandably, a focus on internal attributions leads to consider the self as the primary source of change and thus the primary target of criticism when confronted with difficulties.

By saying “don't count yourself out,” the following participant implied that the problem resides in people not trusting themselves with available opportunities, and so we should “say to parents, higher education today, look how many people go there and say to yourself it's not just for the elites y'know… don't be afraid, don't count yourself out.” (#7). This points to an individual-level barrier that both neglects the influence of structural factors in educational and professional access and sets elitist environments as the golden standard.

Yes it's true that the thesis that really was an ordeal. Umm and I have attended conferences myself on personal development actually [hmm] which really made me realize my limitations. Limitations which I placed on myself and also a great acceptance of responsibility because at the beginning when you don't succeed you tell yourself it's the other person, it's other people, it's other people, it's the context, it's everyone but you don't question yourself and it's a challenge in fact, it's about taking responsibility and so well there you are… taking yourself in hand (laughter). (#22)

This quote directly echoes the work of Rimke (2000) on the self-help literature as a means to expand neoliberal prerogatives and install neoliberal governmentality. Indeed, here assisting to personal development conferences led the participant to bear full responsibility for her struggling throughout her PhD thesis. As entrepreneurial subjects, people are indeed expected to regulate their thoughts, their emotions and behavior and are encouraged to engage in self-discipline, self-surveillance and self-optimization (Foucault, 2004a,b; Riley et al., 2017). More broadly speaking, the entrepreneurial self manages him or herself like a firm (Brown, 2016; Teo, 2018). This entails that people should invest in themselves and their future, in their human capital, always improving and finding new goals (Binkley, 2011a; De La Fabián and Stecher, 2017; Arfken, 2018). When describing their life goals, several participants did report aiming primarily at personal fulfillment and growth (McDonald and O'Callaghan, 2008; Binkley, 2011a; Adams et al., 2019). The following participant expressed this expectation clearly by acknowledging that progress and personal growth were more important than short-lived victories:

For me success is not, it's not being exceptional once for example. […] It's being, it's making progress in fact in my opinion. So as long as I'm going my way, making progress, having small wins, I'm successful. (#9)

Well for me in any case, it's rather a part of personal development… more than financial, I'm not going tell myself I've made a success of my life because when I'm fifty years old I have a Rolex on my wrist. But rather tell myself I've seen this, I've seen that, I've seen this, I've made discoveries, I've learned from people, I've learned from lots of things, and based on that I'll tell myself: okay from my personal point of view my life has been a success. (#7)

The participant above (#7) defined success in life as relating to personal growth rather than material accumulation. However, because selfishness and individualism have a negative connotation in the French normative context (Springer et al., 2016), some participants also insisted that beyond the desire for self-improvement, they also wished to help others increase their human capital. Another participant indeed mentioned the willingness to help others “open their minds and their abilities, well their range of options” (#7), positioning himself as the stepping stone for other people' success.

The participant below mentioned trying to start a business that would offer conferences and lectures on personal fulfillment and growth in order for people to gain more control over themselves. In sum, to be better self-regulators, to reach their goals and be happy. Therefore, behind this genuine benevolence, it is the neoliberal way of being, and more specifically the entrepreneurial self, that this business plan is likely to promote:

So personal development is umm in fact all internal control, so that in fact you're able umm to appreciate these processes, processes which work and which don't work. And umm help people become aware of the way they function and lead them to a different way of functioning which will allow, or bring them well-being, or actually, to refine their objectives and achieve their objectives. (#22)

Therefore, despite their willingness not to be/sound too individualistic, those participants still expand neoliberal prerogatives beyond themselves.

The entrepreneurial subject is also one that maintains a rationale of cost-benefit in many aspects of one's life, including in their relationships (Teo, 2018; Ratner, 2019). This means preserving those that are unequivocally beneficial to the self (Adams et al., 2019; Morris and Korobov, 2020), any other kind of relationship should be disregarded. This leads to utilitarian relationships where individuals become a means to an end. This was explicitly expressed by one participant when quoting a work colleague:

There are things which make social integration easier and the development of a network which is now super important and also when you're at business school that's all you hear about […] I've a colleague who says to you; every day you spend quarter of an hour networking. […] the quarter of an hour networking it's umm. […] you spend a quarter of an hour helping people who need it from your close circle, or a little wider, whether it's looking for work, looking for an apartment, if it's things that interest them you send them a link [ah] it can be things like that (silence). With the aim, behind that, it's self-interest, it's telling yourself; if I've taken time for them, they're bound to take time for me. (#7)

Many participants did mention the importance or necessity of sorting relationships when they are considered as barriers:

If you have friends, but, but… you realize in fact that that doesn't have a lot of value, that you don't really feel any added value spending time with them, that means that in fact you're wasting your time so umm… so that means I'm not doing as well as I'd like because it doesn't actually get me any benefits. (#15)

By saying “you don't really feel any added value spending time with them,” this participant implied that there *should* be an added value to spending time with friends, therefore that is does not hold intrinsic value. One should be able to calculate this added value and act accordingly. Thus, people should extend their social networks to access new opportunities and horizons and thus be continuously enriched:

If these people around you are always the same, if that never changes, you could say that in this respect umm… you're restricting yourself to a specific context. So they hold you back in the sense that if you don't make the effort to, to expand your circle of friends you might say, well you'll stay at… the same level. (#15)

Interestingly and relatedly, several participants reported having no interest in investing work relationships that are perceived as unnecessary, interchangeable and optional. For instance for this participant who mentions important relationships as “friends, family. Colleagues no, I couldn't give a damn. Colleagues, they're only colleagues.” (#32)

The entrepreneurial self is also one that overcomes obstacles and refuses victimhood (Baker, 2010). This implies invoking difficulties of the past more so than present ones (Scharff, 2016), and looking for a silver lining to every hardship. People are invited to look at the bright side, to reconstruct their biographies from a positive perspective, to believe that there exist solutions to every problem, to capitalize on any event, be it positive or negative and to frame difficulties as inspiring, surmountable events (Baker, 2010; De La Fabián and Stecher, 2017), “I know well that there are times when you have… things that you lack or whatever. You'll try to get the best out of every situation you'd say.” (#30)

As another example, the participant below reframed an economic lay-off as “best luck” because it gave him the incentive to start a new project:

Well that's life, it gives you opportunities… If you manage to seize them umm, […] I'd say that the best luck I had was an economic lay-off. That allowed me to get going. I took a gamble on it and… It worked. (#13)

In sum, entrepreneurial subjects should self-regulate and invest in themselves, with time, energy and money, to adapt, to learn new skills in order to succeed in selling themselves in the competitive market (De La Fabián and Stecher, 2017; Arfken, 2018).

So far, we have seen that part of neoliberal ideology and its ability to abstract people from their context, consisted in shaping individuals as responsible, calculative, self-regulated and autonomous agents. Importantly, the promotion of self-regulation was found to extend to emotional responses.

### Emotional Management

Several participants expressed the importance of being in control of their inner state and mood, which once again, fuels the abstraction from context mentioned before. For instance, the following participant wished through her professional activity, to help people first be aware of their emotional receptivity to their context and then gain their freedom and independence through the mastering of this receptivity. In so doing, she implicitly framed inner emotional changes due to external causes as something negative and enslaving. Through self-help conferences, she thus wished to offer guidance for self-control and therefore freedom:

Most people don't realize that its external conditions that influence their mood and their state of being and also that's the aim of my approach and my conference. It's actually becoming aware of this and actually allowing people to acquire their freedom and independence. (#22)

Anger and negative emotions in general are expected to be down-regulated (Scharff, 2016; Adams et al., 2019) in a neoliberal framework that upholds happiness and well-being. For instance, a participant described anger as being a barrier to the accomplishment of her entrepreneurial self: her being productive, because “you can't be creative in that state [being angry] in fact” adding that what could “stop [her] being free is that [she's] actually going to get into states of mind which are not productive.” (#22)

Neoliberal ideology indeed enhances the quest for positive emotions, imposing the happiness diktat and positing that being unhappy is tantamount to failure (McDonald and O'Callaghan, 2008; Binkley, 2011b). We did find that happiness was an important, spontaneously recurrent theme in participants' discourses. Many participants reported that their “main wish is to be happy” (#1), to aim for “personal development. [Eh eh] that's, well that's a success in life, well umm… I think that applies to all fields.” (#11)

Interestingly, many participants who expressed that wish shared the impression that it was counter-normative. Thus and related to the abstraction from normative context theme, neoliberal ideology seems to have succeeded in creating an illusion of uniqueness while leading people to pursue normatively circumscribed goals (Bröckling, 2005). For instance, the participant below contrasted perceived normative goals oriented toward financial success with her “own” goal of being happy:

Society wants me to succeed in life by having a good job and having a good financial situation, etc. personally it's not necessarily the same thing in fact, it's not necessarily the same definition. For me it's when I'm happy in my life generally. (#26)

Besides being accomplishments in themselves, positive emotions such as happiness also become clues that one is succeeding. It is not only a goal but also an indicator that one is on the right track:

I think that succeeding in life is umm… it's feeling happy where you are, and… fulfilling yourself and being, not necessarily anything to do with criteria and objectives and umm [huh] yeah, it's rather to do with that and… and me, I do something I like umm… and I'm pretty happy umm (#29)

Going even further, the next participant placed individual satisfaction as the preferred criteria for the distribution of resources. According to this participant, resources can be unequally distributed as long as people are satisfied with what they get. This echoes work by Kay and Jost (2003) on complementary stereotypes of “poor” but “happy” as a means to justify social inequalities:

Human beings, at bottom, by nature, are not equal but all the same it's up to society to act in such a way that all choices can lead, it's not even a question of equality or anything it's more hmmm about satisfaction. If I am satisfied with… 1+1 and someone else is satisfied with 1+2, er so be it. Well it's umm…But it's a form of equality because the two don't adopt the same figures but will be equally satisfied. (#5)

At odds with this pursuit of happiness is the fact that neoliberal ideology may also trigger feelings of anxiety through the focus on personal responsibility and the increased perception of choice (Baker, 2008, 2010). In fact, neoliberal ideology has been described as an anxiety-oriented ideology (Mcdonald et al., 2007; Anderson, 2015). Neoliberal practices and expectations may indeed exacerbate the pressure of choosing right, being always happy, winning the many competitions people are confronted with and more generally of being at the top of one's game. In the interviews, we did find many reports of stress or anxiety. Understandably and as observed in the “competition” section, work environments that highlight competition or productivity generated stress among participants.

In sum, while neoliberal ideology comprises high expectations and standards of success, including at being happy, it also increases the chances of falling short of those expectations. In leading people to disavow social support and in highlighting personal responsibility, it becomes hard and lonely to reach neoliberal prerogatives. Moreover, as illustrated above, competition also represents a major source of stress in modern societies where people are competing at school, for a job, a promotion, for sports, friends and so on. Additionally, neoliberalism has been associated to increased precariousness and instability on the job market (Dardot and Laval, 2010), and there is evidence that poverty, inequalities, unemployment or social isolation are related to higher rates of common mental disorders such as depression and anxiety, which have been on the rise in the past decades (Rose, 2019). This inflation may indeed stem from increasingly unequal societies, and from a decrease in welfare provisions in many countries of the world (Rose, 2019). Those changes may therefore thwart people's efforts and possibilities to actually reach and maintain those prescribed positive emotional states. Therefore, neoliberal subjects do strive for happiness and learn to self-regulate their emotions, but the neoliberal reality, through its practices and norms, does not necessarily facilitate the actualization of its own injunctions.

## Discussion

The main purpose of this study was to draw the contours and content of neoliberal ideology in France (i.e., a case of historical statism). Relying on a template analysis of 32 interviews and on the existing literature, we established five central themes characteristic of this ideology: (1) State prerogatives, (2) Competition, (3) Abstraction from context, (4) the Entrepreneurial Self, and (5) Emotional management. Importantly, those themes combine elements seemingly “apolitical” such as a focus on self-regulation and happiness, and elements outwardly political such as the role of the State in France.

### Neoliberal Common Ground

We found numerous similarities between the content of neoliberal ideology as described in the literature and as found in our interviews. Those similarities concerned the relative abstraction from the social and institutional structures, which includes the way participants link social inequalities to individual-level variables such as ignorance or lack of merit, and which also transpires in the marginal role the institutions play in the reproduction of social inequalities in the eyes of participants. Those similarities also concerned the endorsement of meritocratic values and the perception of competition as a necessity and major source of motivation. Moreover, we have seen that the notion of inequality often took on an apolitical meaning and in fact, a depoliticization of inequalities have been described in the literature as a consequence of neoliberal ideology (Brown, 2015; Arfken, 2018). Finally, those interviews highlighted the construction of an entrepreneurial self that focuses on self-improvement, happiness and on building and profiting from its human capital.

### Localized Variations

However, beyond those similarities, we also found ways through which neoliberal ideology adapted to French social norms, values and institutions. For instance, we have seen that the negative connotation of individualism in France led participants to refuse the idea that they should only care about their own human capital. Indeed, while they largely value independence, they also recognize the importance of social ties and express a willingness to help others increase their human capital as well. Also and importantly, most participants don't want to see the State completely disengage from its social duties. Additionally, we have seen that participants rationalized inequalities only to some extent. Neoliberal ideology has to make its way around or through a norm of equality, and it partly does. Indeed, while participants expect the State to fight inequalities of opportunity, they still sometimes disavow the term “equality” and show little consideration for equality in resources.

Importantly, advocating for equality in opportunity is a lot less threatening to the neoliberal order than demanding equality in resources because it preserves the necessity of -a fair- competition that would reward people depending on their individual merit and because it does not question income disparities. Thus, we can see that this “equality norm” can still be, at least partially, reshaped and adapted to neoliberalism (e.g., Sénac, 2017).

Eventually, the shift in the definition and focus of “equality” (i.e., equality of opportunity vs. equality of resources) may well downplay a form of normative and historical resistance to inequality (Duru-Bellat, 2019), and resistance to inequality is potentially a resistance to neoliberalism and capitalism more generally. In sum, several French variations and differences that we observed did not seem to be that far off neoliberal ideology.

### Places of Resistance to Neoliberal Ideology and Neoliberalism

Interestingly, we also found tensions and contradictions between certain neoliberal prescriptions themselves, and with French social norms or actual experiences of participants. For instance, while neoliberal ideology promotes competition, it also promotes positive emotions and happiness. For some participants, those two elements are not compatible. Moreover, several participants found themselves uncomfortable in the gap between the extreme definition they give of notions such as independence, their willingness to consider themselves as independent individuals and their actual social interdependences. Similarly, we have seen that at times, neoliberal injunctions ran against French norms such as the expectation of a certain degree of equality and the negative connotation of individualism. For instance, the expansion of competition to close social relationships seems at odds with French norms against individualism. We posit that in such inconsistencies, resistance to neoliberal ideology may form and develop. Likewise, the French's general attachment to the welfare State can hinder, or slow down, the extension of neoliberalism and the privatization of State-run services. Moreover, crises like the one caused by the coronavirus (that started at the end of 2019) may well-highlight the advantage of having a strong public health care system and a strong State in general to support the threatened economy, and may render the neoliberal rhetoric of austerity and State retrenchment even more difficult to articulate.

### Limits of the Study

Certain limitations can be highlighted in this study. First, our findings are necessarily contingent on the experiences of those 32 participants and their level of exposure to neoliberal ideology. There probably exist over and/or under representations of certain aspects of neoliberal ideology that are related to the composition of our sample. Evidently, all aspects of the results may not be transferrable to other countries and populations. Also and relatedly, because our objective was to identify themes and specificities of neoliberal ideology in France, the present analysis only focused on the detection of such elements and not on sub-groups comparisons concerning the prevalence of specific aspects of the ideology. This could also be useful in order to better understand which aspects of the ideology seem more attractive to or are more directly targeted at specific sub-groups, and how the norms of those sub-groups interact with the neoliberal diktat. For instance, women have been described as the primary target of messages of self-transformation, notably through bodily improvements and alterations (Bröckling, 2005; Rutherford, 2018), which may add on norms of femininity.

More research is thus necessary to counter those limitations and to further our understanding of neoliberal ideology.

### Implications

We believe that the present research can help nourish several research fields in psychology. For instance, it has been shown that an important implication of neoliberal ideology is that it diminishes the chances of one engaging in collective action (e.g., Girerd and Bonnot, 2020; Girerd et al., 2020). In our interviews, we did find elements that help us understand this phenomenon. Indeed, the importance of the self as the source and target of change, the definition of independence as the ability not to rely on other people, the quasi absence of a sense of duty toward one's group may lead people to think that there is nothing to expect from collective action. Moreover, engagement in collective action requires that one feels angry about the status quo (van Zomeren et al., 2008; Becker and Tausch, 2015), and instead, neoliberal ideology seems to be more anxiety-oriented (Anderson, 2015; Scharff, 2016). Similarly, the belief in a meritocratic distribution of resources serves system justification purposes (e.g., Jost and Hunyady, 2005; Darnon et al., 2017). Indeed, it stands as an ideological tool to explain and rationalize people's position in the social hierarchy in invoking individual merit. Those qualitative results can thus help understand how people describe and rationalize inequalities in a French context.

Relatedly, this study helps us reflect on current work relationships and collective action in the workplace. Indeed and interestingly, few participants expressed an interest in investing work relationships. This may be related to increasingly mobile and flexible work environments and to the increased probability of workers to be in and out of the workforce and to experience many work environments throughout their career (Hélardot, 2005; Lallement, 2008). This may have dire consequences, notably in workplace solidarity and union participation. Combined with the diffusion of teleworking recently accentuated because of the confinements due the COVID-19 crisis, it may diminish workers' collective abilities to resist and oppose oppression or exploitation in the workplace. This may even be more problematic for minorities who may find it difficult to address injustice in the workplace while being and/or feeling isolated. It echoes Bourdieu (1998)'s argument that neoliberalism is in fact a “program of destruction of collective structures.”

Finally, while focusing on the silver lining in the face of hardships may allow for greater psychological well-being, the downfall may be twofold: first, people may feel guilty and/or be criticized if they don't live up to the expectation of being constantly happy and resilient, and second, even oppressive and exploitative inter-group relations can be looked upon from a positive perspective, once again turning people away from the collective action that could redress injustice (Baker, 2010). Moreover, the efforts one can deploy for one's happiness are now increasingly enabled by scientific developments (e.g., neuroscientists collaborating with software and app developers) and measurements as well as data-driven, personally targeted advertisements that lead to a marketisation of happiness (Davies, 2016). Happiness becomes another form of human capital within the “happiness industry” (Davies, 2016). This fits with the neoliberal framework that often brings momentary individual-level reassurance and well-being but that hinders systemic changes and collective solutions (Teo, 2018).

This study could therefore also be useful for research on coping strategies, emotion regulation and the palliative effect of ideologies (e.g., Napier et al., 2020), as well as in health psychology more generally (Becker et al., 2021).

## Conclusion

Endorsing a qualitative approach has allowed us to build a rich and complex image of neoliberal ideology in France (i.e., a case of historical statism) highlighting both similarities and differences with what can be commonly found in the literature. We have indeed found evidence of a neoliberal governmentality going beyond State control in order to have citizens shaping themselves to meet neoliberal and capitalist demands of commodification, self-investment, self-regulation—thus alleviating State obligations—and be competitive and hyper-productive. However, the present results also highlight the necessity to consider cultural and structural specificities when trying to study hegemonic ideologies such as neoliberal ideology.

In sum, while neoliberal policies originally came from a few influential think tanks, their influence now seems to have expanded to people's very psychology. The tensions, contradictions and resistances that arise in people's discourses do not seem to prevent neoliberal ideology's expansion.

## Data Availability Statement

The datasets presented in this article are not readily available because we did not ask participants for their consent to share the data contained in the interviews. Requests to access the datasets should be directed to lola.girerd@u-paris.fr.

## Ethics Statement

Ethical review and approval was not required for the study on human participants in accordance with the local legislation and institutional requirements. Written informed consent for participation was not required for this study in accordance with the national legislation and the institutional requirements.

## Author Contributions

LG and VB contributed to the research design. LG did the interviews and wrote the first draft of the manuscript. LG, VB, and CV contributed to the data analysis. All authors contributed to the recruitment of participants, manuscript revisions, read, and approved the submitted version.

## Conflict of Interest

The authors declare that the research was conducted in the absence of any commercial or financial relationships that could be construed as a potential conflict of interest.

## Footnotes

^1^For work describing a shift from a liberal to a neoliberal tradition, see for instance Simon (2016) and Foucault (2004a).

^2^Verniers, C., Bonnot, V., and Assilamehou-Kunz, Y. (submitted). Intensive mothering and the perpetuation of gender inequality: evidence from a qualitative study of mommy blogs. [Manuscript Submitted for Publication].
