# Supplementary material for: Neoliberal Ideology in France: A Qualitative Inquiry
Source: Front Psychol. 2021 Jun 29;12:686391. doi: 10.3389/fpsyg.2021.686391 (PMC8276107; doi:10.3389/fpsyg.2021.686391)
Supplement: Supplementary file 1 [file Table_1.DOCX]

**Supplementary Material 1**

*Themes and sub-themes of analysis, their definition as well as total number of occurrences for each sub-theme*

| **Themes** | **Sub-themes** | | **Definition** | **Number of occurrences** |
| --- | --- | --- | --- | --- |
| **State prerogatives** | Desire for equality | belief-society=desire for equality | Perception that there is a willingness from politicians or/and people in general to have a more egalitarian society. Perception of a norm of equality. | 44 |
|  |  | school-desired role=to enhance equal opportunity | The education system should ensure that for the next stages of life, people have equal opportunities, that everyone should have the opportunity to reach certain jobs, especially elitist ones after their passing though the education system. | 22 |
|  |  | State-intervention=public interest | Perception that the State must intervene when public interest in concerned: to reduce inequalities, run public services... | 179 |
|  | Perception of inequalities | inequalities=denunciated | Inequalities are being denounced, criticized, are perceived as not normal or/and unfair. | 183 |
|  |  | inequalities=normal | Inequalities between individuals or groups are perceived as normal, fair, sometimes amount to mere inter-individual differences. | 60 |
|  |  | inequalities-description=in terms of opportunities | The participant describes inequalities in terms of opportunity, of access to resources | 154 |
|  |  | inequalities-strategies=should be meritocratic | Mention that strategies to reduce inequalities should respect the merit principle (e.g., opposition to quotas) | 18 |
|  | Resources distribution | desired-resources-distribution=meritocratic | The participant expresses a preference for a resources distribution that is based on merit. | 8 |
|  | privatization | privatization=ambivalence | Perception of both positive and negative aspects of privatization, sometimes due to the fact that the participant does not know what is public or private, or does not understand the implications of privatization. | 24 |
|  |  | privatization=reduces costs | Perception that privatization leads to cheaper services/or reduces the cost of production for those services (i.e., cheaper for the customer and/or the State) | 5 |
|  |  | privatization=efficacy/ quality | Perception that privatization allows for more efficiency, thus to the realization of more goals in less time. That it would also allow for services of better quality. | 16 |
|  |  | privatization=unequal | Perception that privatization will or does widen existing inequalities, in terms of access to those services that will become more limited for people with lower income… | 17 |
|  |  | privatization=positive | Perception of positive aspects, upsides of privatization | 15 |
|  |  | privatization=negative | Perception of negative aspects, downsides of privatization | 43 |
| **Competition** | Beliefs about competition | competition=inevitable | Perception that competition is inevitable, that no one can escape it, either because it is perceived as part of human nature, or because it is imposed everywhere. | 21 |
|  |  | competition-field=personal | The participant describes competitive relationships with friends or closed ones, in activities that are non-professional/academic/sports related | 28 |
|  |  | competition= individual position | Perception that competition is a personal choice, or the result of personality trait. People put themselves in competitive relationships or contexts. | 55 |
|  |  | competition= with oneself | Perception that one is competing with oneself, in a desire for self-improvement... | 6 |
|  | Effects of competition | competition-upsides=for individuals | Perception of upsides of competition for individuals themselves, in terms of motivation, increase in performance and development of skills, individual progress. | 58 |
|  |  | competition-downsides=for individuals | Perception of downsides of competition for individuals themselves, in terms of self-esteem, stress, feelings of inferiority. | 37 |
| **Abstraction from structural context** | Perception of inequalities | inequalities-source=within individuals | Inequalities are perceived as emanating from individual characteristics and are often described at an inter-individual level (e.g., related to a lack of confidence, health issues, difficulties to adapt...) but also at a group level (e.g., racism due to ignorance or cognitive bias). | 55 |
|  |  | inequalities-types=individual | Inequalities are described at an inter-individual level, in terms of differences in physical, intellectual abilities... | 73 |
|  |  | inequalities-strategies=individual | Strategies to reduce inequalities should rest upon individuals themselves, through trainings, effort, self-alterations, personal choices... | 41 |
|  |  | inequalities-change=individuals | Perception that changes to reduce inequalities should be directed toward individuals, changing their mentalities, behavior, thoughts, emotions, in opposition to changing the societal structures. | 91 |
|  |  | inequalities=normal | Inequalities between individuals or groups are perceived as normal, fair, sometimes amount to mere inter-individual differences. | 60 |
|  | Source of obstacles | obstacle-attribution=internal | Obstacles to advancement in life, success... are perceived as emanating from individuals themselves: lack of skills, self-confidence, health issues, bad self-regulation... | 140 |
|  | Definition of independence | independence-definition=in terms of loneliness/ detachment/self-sufficiency | Independence is defined as the ability to bear loneliness, to like having "alone" time, to need very few people, to be self-sufficient in terms of closed others and societal structures | 85 |
| **Abstraction from normative and social context** | Perception of dependence | dependence=depreciated | Dependence, however defined, is depreciated, described as something negative, harmful, something to be avoided. | 18 |
|  |  | dependence=distancing | The participant does not want to be perceived as someone who is dependent upon others, she/he tries to distance her/himself from this image in reaffirming her/him independence on certain aspects. | 3 |
|  |  | dependence=expression of individuality | The participant speaks of relational dependences as something that is related to a personality trait, something characterizing her/himself: negation of a widely shared desire for meaningful social connections. | 12 |
|  |  | dependence=vulnerability | Description of social dependences as sources of vulnerability or weakness. | 6 |
|  | Perspective on social norms | norms=detached | The participant feels detached from the social norms she/he is mentioning, without necessarily disagreeing with them, she/he doesn't perceive her/himself as being constrained by those norms. | 47 |
|  |  | norms=rejected | The participant expresses a disagreement with certain social norms and a willingness to oppose them. | 43 |
|  | Definition of independence | independence-definition=absence of external influence | Description of independence as the ability to be counter-normative through one's behavior, thoughts, way of life... ability to extract oneself from normative pressures, not to let oneself be influenced by external factors, the media, other people... | 68 |
|  |  | independence-definition=in terms of loneliness/ detachment/ self-sufficiency | Independence is defined as the ability to bear loneliness, to like having "alone" time, to need very few people, to be self-sufficient in terms of closed others and societal structures | 85 |
|  | Sense of duty/obligation | duties=citizen/man/ human/ parent/employee/boss | The participant mentions obligations or duties that one has towards society, towards their children, their employees or boss, or duties one has as a human being towards others. | 12 |
|  | Free choice | choices=necessitate support | The participant expresses the need to rely on others when confronted to a choice to make. | 7 |
| **Entrepreneurial self** | Goals | goal-attainability=endlessly renewable | Perception that goals are endlessly renewable, to one always has (to have) new goals to reach. | 23 |
|  |  | goal-target-others=increase their human capital | The participant expresses the goal to help others increase their human capital, to discover new things, to be better self-regulators, to be a steppingstone for people's own success. | 5 |
|  |  | goal-target-self=cultural capital/skills | The participant expresses the goal to acquire new knowledge, new skills, to evolve. | 30 |
|  |  | goal-strategy=self-transformation | Perception that one can achieve their goals through self-transformations, self-surveillance, self-regulation, more personal effort, a change in perspective one has on a specific matter. | 134 |
|  | Perspective on obstacles | obstacle-temporality=in the past | The participant speaks of difficulties of the past. | 35 |
|  |  | obstacle=inspiring/ surmountable | The participant speaks of obstacles or hardships as surmountable events, they mention positive things that one can draw from difficulties that are described as sources of learning, enrichment, as something inspiring rather than discouraging or unsurmountable. | 53 |
|  |  | obstacle-attribution=internal | Obstacles to advancement in life, success... are perceived as emanating from individuals themselves: lack of skills, self-confidence, health issues, bad self-regulation... | 140 |
|  | Definition of independence | independence-definition=associated with risk taking | Independence is described as related to risk taking. | 11 |
|  | Perspective on social relationships | relationships with others=utilitarian | Relationships are established with a specific goal in mind, to find a job, to obtain promotions... Relationships described as means, not as ends, notion of cost/benefits. | 16 |
|  |  | relationships with others=conditional/ relational mobility | Expression of a choice in the creation, maintenance or dissolution of social relationships. Expression of the distinction between different types of attachments, to set limits in certain relationships, expression of a disengagement from certain collective solidarities, of a choice in the degree of social engagement. | 61 |
| **Emotional management** | Emotions | expressed emotion=fear/ anxiety | The participant expresses fear, worry or anxiety over a particular topic. | 42 |
|  | Goals | goal-target-self=to be happy | The participant expresses a willingness to be happy, to feel good, to have positive emotions. | 85 |
|  |  | goal-attainability=emotional clues | Perception that one knows she or he has reached their goal through emotional clues, a feeling of satisfaction, of accomplishment, of well-being despite the fact that the goal itself was not explicitly to be happy. | 44 |
|  | Relationship with others | relationships with others-negative=psycho/ emotions | Description of relationships as potential sources of negative experiences, criticism, insults... People can cause negative emotions, stress, anger... | 33 |
|  | Resources distribution | resources-distribution=for happiness | Resources should be distributed so that people be happy, could do what makes them happy. Therefore, the amount of resources distributed is irrelevant as long as people are happy with what they have. | 4 |
|  | Free choice/freedom | choices=source of anxiety | Decision making is described as a source of anxiety, or other negative emotions. | 2 |
|  |  | freedom-definition=in terms of self-control | Freedom is defined as the ability to control oneself, not to be determined by one's emotions, dependences... | 13 |

*Note*. This table aims to complement the information present in the main text, by showing the subthemes that we relied on as well as their definition and number of occurrences. This allows to have a quantitative overview of the prevalence of each subtheme, and to know how we compartmented our analyses.

**Supplementary Material 2**

*Complementary analyses: State prerogatives*

Interestingly, several participants did not have a clear comprehension of what the French public services were, nor of what privatization entailed. It may reflect that French people are ill-educated about their own public services:

It’s true that that [privatization], that was the thing with SNCF. [Yes, for example] I haven’t understood everything, I have to admit that I don’t know at all umm ... what’s good and what’s not good. [...] No I admit that I don’t realize umm ...I don’t realize what that would do, what that would change, both for the state and for us. (#28)

*Complementary analyses: Competition*

Another illustration of participants’ distanciation from a perceived competitive mindset can be found in the way the following participant expressed its futility “already if you want to have a better house than your neighbor, a prettier garden, bigger tomatoes. I think that everyone can see competition behind everything they want y’know but well umm personally I don’t see the point.” (#3)

A desire for endless improvement also brought some participants to report competing with themselves, not only with others, as was observed in Scharff (2016), “in fact I’m more in competition with myself than with other people. I have to succeed for myself, I don’t have to umm, well I don’t have to succeed for other people.” (#32)

*Complementary analyses: Abstraction from structural contexts*

Besides leaving the institutional structures off the hook, blaming ignorance or selfishness for inter-group inequalities also affects the perceived temporality of inequality reduction, because educating people takes time:

I don’t see what you could do, apart from trying to change attitudes, but well that, that would take 20-30 years. We’re making progress, it’s really good there’s no worries, we’ll get there gradually definitely, but overnight that’s not possible in my opinion. Even over a relatively short period, it’s not possible. (#2)

This participant’s comment implies that we should not expect too much too fast from the State nor from collective action because changing mentalities takes time.

*Complementary analyses: Abstraction from normative/social contexts*

Depending on other people can also be seen as a source of vulnerability in the sense that those we rely on might not always be there. Thus, without appearing as a necessity, being self-dependent stands as a precaution:

There are some people who are quite happy to live their life umm... without being independent. On the other hand, they're going to feel a bit funny when the people they depend on are no longer there. So it’s important to be independent to prepare for that moment. (#7)

Interestingly and consistent with Baker (2008)’s findings, neoliberal ideology’s focus on free choice also creates new pressures. Indeed, navigating through -what neoliberal ideology describes as- an ocean of choices and opportunities creates a new expectation that people must make good decisions. Understandably people often seek social support to make decisions but they might now feel guilty for doing so because it seems at odds with the notion of self-reliance (Baker, 2008):

It could be couple or anyway romantic decisions yes, it could be hey should I umm... I’d do that rather than that, even, even with my train tickets, kinda like, hey do you think I should return to Paris on Sunday evening or Monday morning, it’s things like that, I have a great need for reassurance. And sometimes it’s a bit stupid because, you tell yourself umm, I feel reassured but in relation to…always to some sort of norm. (…) Your reaction isn’t based on yourself. Well in any case to the, the will you have inside you. (#7)

While admitting that he needs reassurance and advice to make certain choices, this participant still depreciated this need because according to him, choices should reflect one’s own personal desires and no one else’s.

We found another illustration of this abstraction from context in the noticeable absence of notions like “duty” and “social obligations” in participants’ discourses. This is consistent with Nafstad et al. (2007)’s observation that the terms “duty/obligation” in Norwegian newspapers’ had been decreasing from 1984 to 2005, following the expansion of neoliberal ideology. Indeed, in a neoliberal framework, abstraction from context not only means omitting external influences on our personal and group experiences, but it is also refusing the burden of social obligations and collective responsibilities that come with our embedment in a social environment (Bettache et al., 2020; Nafstad et al., 2007).

*Complementary analyses: Entrepreneurial self*

Developing the entrepreneurial self and increasing one’s human capital sometimes require taking risks. Indeed, neoliberalism has been described as a system that promotes risk taking, risky investments rather than inaction (Gershon, 2011; Harvey, 2007; Salzinger, 2020; Scharff, 2016). In the same vein, several participants mentioned that choosing to be independent was sometimes a risky move and they described it in a positive light:

Financial independence I think that err it's [...] being able umm... allowing yourself to take risks, to attempt [...] to go and try and achieve the objective you have set yourself and umm.... it’s partly the freedom to take risks umm...because actually you are, you are sufficiently – it's independence a bit y’know well you are sufficiently .... secure to allow yourself to go [huh] and do what you want. (#29)

*Complementary analyses: Emotional management*

Considering happiness as a guiding principle also applies to social relationships. If you don’t “feel” good with someone, then you should disregard this relationship, as expressed by the following participant:

Someone who holds me back umm I’d get rid of them very quickly umm [hmmm] from my close circle. I’ve noticed that I’m driven by feeling, when that doesn’t work with someone well I break away because actually umm I’ve always been told to avoid staying with people who drag you down” (#18)
